# Variable-Fidelity Simulation Models and Sparse Gradient Updates for Cost-Efficient Optimization of Compact Antenna Input Characteristics

**DOI:** 10.3390/s19081806

**Published:** 2019-04-15

**Authors:** Slawomir Koziel, Anna Pietrenko-Dabrowska

**Affiliations:** 1 School of Science and Engineering, Reykjavik University, 101 Reykjavik, Iceland; koziel@ru.is; 2 Faculty of Electronics, Telecommunications and Informatics, Gdansk University of Technology, 80-233 Gdansk, Poland

**Keywords:** antenna design, internet of things, surrogate-based optimization, trust-region framework, variable-fidelity EM simulations

## Abstract

Design of antennas for the Internet of Things (IoT) applications requires taking into account several performance figures, both electrical (e.g., impedance matching) and field (gain, radiation pattern), but also physical constraints, primarily concerning size limitation. Fulfillment of stringent specifications necessitates the development of topologically complex structures described by a large number of geometry parameters that need tuning. Conventional optimization procedures are typically too expensive when the antenna is evaluated using high-fidelity electromagnetic (EM) analysis, otherwise required to ensure accuracy. This paper proposes a novel surrogate-assisted optimization algorithm for computationally efficient design optimization of antenna structures. In the paper, the optimization of antenna input characteristic is presented, specifically, minimization of the antenna reflection coefficient in a given bandwidth. Our methodology involves variable-fidelity EM simulations as well as a dedicated procedure to reduce the cost of estimating the antenna response gradients. The latter is based on monitoring the variations of the antenna response sensitivities along the optimization path. The procedure suppresses the finite-differentiation-based sensitivity updates for variables that exhibit stable gradient behavior. The proposed algorithm is validated using three compact wideband antennas and demonstrated to outperform both the conventional trust region algorithm and the pattern search procedure, as well as surrogate-based procedures while retaining acceptable design quality.

## 1. Introduction

Design of antennas for the Internet of Things (IoT) poses specific challenges. These include the necessity of integrating the structures into various objects [1], maintaining low profile and low cost. Other requirements are dependent on particular applications and may include: (i) multi-band or wideband operation [2,3,4], (ii) excellent matching (e.g., for energy harvesting [5]), (iii) multiple-input multiple-output (MIMO) functionality to ensure transmission channel capacity (e.g., for high data rate wireless access points and mobile users [6,7]), as well as (iv) close-to-isotropic radiation pattern (e.g., to maintain orientation insensitive communication [8]. Another important requirement, common to the majority of IoT applications, is a small size of the device [9,10]. This is a serious issue because the reduction of the antenna footprint normally results in degradation of both electrical and field characteristics. In order to maintain small size and acceptable performance, various topological modifications are being incorporated into the antenna structures which leads to more and more complex designs [11,12,13] described by a large number of geometry parameters. Appropriate tuning of these parameters is a difficult and time-consuming process, especially when conventional methods, such as parameter sweeping guided by engineering experience, are employed. Considerably better results, including effective control over several performance figures and constraints, can be achieved through rigorous numerical optimization. The bottleneck of either approach is a computational cost, which is generated by massive electromagnetic (EM) analyses associated with the use of standard optimization algorithms, especially the global ones (e.g., evolutionary algorithms [14], particle swarm optimizers [15], but also recent population-based metaheuristics [16,17,18]).

Efficient numerical algorithms are fundamental to make antenna optimization more practical in computational terms. There has been a considerable research effort directed towards this objective, and several promising techniques have been developed. Perhaps the most straightforward approach is the utilization of adjoint sensitivities [19,20] to accelerate gradient-based algorithms over the versions involving numerical derivatives (estimated through finite differentiation) [21]. This is, however, not widespread due to the limited accessibility to the adjoint technology through commercial EM solvers [22]. An alternative is to replace the expensive high-fidelity EM model by a faster representation (or a surrogate model) of the system under design, which is a keystone of so-called surrogate-based optimization (SBO) techniques [23,24]. SBO uses the surrogate as a prediction tool that guides the search process towards the optimum design at a lower computational cost as compared to doing so directly at the level of the high-fidelity model. There are various types of surrogates available that can be roughly split into two groups: data-driven models [25] and physics-based ones [26]. The data-driven surrogates are simply constructed by approximating sampled high-fidelity data, and the popular techniques include kriging interpolation [27], polynomial regression [28], support vector regression [29], or neural networks [30]. These models are generic and fast but not suitable for handling complex antenna structures due to the curse of dimensionality (rapid growth of the number of training data samples required to build the model as a function of the number of parameters) and high nonlinearity of the antenna responses. Practical applications are often limited to rather low-dimensional cases [31,32]. Physics-based SBO methods construct the surrogates using underlying low-fidelity models (such as equivalent circuits in case of microwave components [33]). This makes them less generic but also more immune to dimensionality issues. Unfortunately, fast low-fidelity models are rarely available for antenna structures: the only versatile way of creating these is coarse-mesh EM simulation. Relatively expensive low-fidelity models do not allow for the efficient use of some well-established SBO techniques, such as space mapping [34], but leave some room for other variable-fidelity approaches, e.g., response correction methods [35,36].

In this paper, a novel algorithm for cost-efficient optimization of electrically small antennas, including antennas for IoT applications, is proposed. Our methodology exploits the SBO concepts in the form of variable-fidelity EM simulation models as well as a suitably modified version of the trust-region gradient search algorithm with numerical derivatives. The major mechanisms developed to reduce the computational cost of antenna sensitivity updates through finite differentiation are (i) estimation of the response derivatives at the level of a low-fidelity model as well as (ii) monitoring of the gradient stability in the course of the optimization process. The latter permits us to suppress finite differentiation for selected antenna parameters. The resulting algorithm is validated using a benchmark set of three compact wideband antennas and demonstrated to yield considerable computational savings as compared to both the reference trust-region algorithm and its accelerated versions working at the level of high-fidelity EM model, along with the pattern search procedure [37]. The savings are obtained without compromising the design quality in a significant manner. The remaining part of the paper is organized as follows. Section 2 introduces the proposed optimization framework. The outcome of numerical validation and benchmarking as well as discussion of the results are provided in Section 3 and Section 4, respectively. Section 5 concludes the paper.

## 2. Expedited Antenna Optimization Using Variable-Fidelity Trust-Region Search with Gradient Monitoring

In this section, the antenna design problem is formulated as a nonlinear minimization task, and the trust-region gradient search algorithm is recalled as a basis of the optimization framework proposed here. A few comments are also made about variable-fidelity antenna models. The main part of the section is devoted to an exposition of the variable-fidelity gradient search procedure with gradient change monitoring. The comprehensive numerical verification of the method and the benchmarking are provided in Section 3.

### 2.1. Antenna Design as Optimization Task

As briefly discussed in the introduction, the design of antenna structures requires handling various performance figures and constraints. If numerical optimization routines are to be employed in the process, it is mandatory to formulate the cost function that quantifies the quality of the design. In the case of single-objective algorithms, by far the most popular and convenient to use, the cost function should be scalar. For this work is concerned with the development of an optimization framework that exhibits certain features, specifically, computational efficiency, a simple yet frequently addressed task of matching improvement is considered. The problem is to minimize the antenna reflection coefficient within a frequency range of interest, corresponding to the intended operating ranges of the structure. The cost function can be defined as

(1) $$U(x)=\underset{f\in F}{\max}|S_{11}(x,f)|$$

In (1), the vector ***x*** represents the tunable parameters of the antenna, whereas *f* is the frequency within the frequency range of interest *F*. The reflection coefficient *S*_11_(***x***,*f*), evaluated through high-fidelity EM analysis is shown to be explicitly dependent on both parameter vector ***x*** and frequency *f*. For the UWB antenna structures considered in Section 3, *F* is a continuous range from 3.1 GHz to 10.6 GHz.

With the use of (1), the design task may be formulated as

(2) $$x^{*}=\underset{x}{\min}U(x)$$

which is a minimax problem, with *U* being the cost function defined by (1). This formulation is a popular way of handling many performance figures pertinent to antennas (e.g., minimizing in-band gain variability and reducing sidelobes).

A popular optimization technique utilized to solve (2) in a local sense is discussed in Section 2.2. Section 2.4 introduces its accelerated version proposed in this work, partially based on variable-fidelity EM simulation models (cf. Section 2.3).

### 2.2. Trust-Region Gradient Search

The reference algorithm is the conventional trust-region (TR) gradient-based routine (e.g., [38]). It solves the problem (2) in a local sense by producing a series of approximations to x^*^, denoted as ***x***^(*i*)^, *i* = 0, 1, … These are obtained by optimizing a linear expansion model *S_L_*^(*i*)^ of the reflection coefficient *S*_11_(***x***,*f*) defined at the parameter vector obtained in the *i*-th algorithm iteration ***x***^(*i*)^ as

(3) $$S_{L}^{\left(i\right)}(x,f)=S_{11}(x^{\left(i\right)},f)+G_{S}(x^{\left(i\right)})\cdot (x-x^{\left(i\right)})$$

In (3), ***G****_S_* denotes the reflection gradient i.e., the antenna reflection *S*_11_(***x***,*f*) sensitivities w.r.t its parameters. We have

(4) $$x^{\left(i+1\right)}=\arg\underset{x;-d^{\left(i\right)}\leq x-x^{\left(i\right)}\leq d^{\left(i\right)}}{\min}U_{S}(x)$$

The objective function *U_S_* in (4) is defined as

(5) $$U_{S}(x)=\max\{f\in F:S_{L}^{\left(i\right)}(x,f)\}$$

i.e., it is an equivalent of (1) at the level of linear expansion model *S_L_*. Typically, the gradient ***G****_S_* is evaluated through finite differentiation, which is the major contributor to the computational cost of the optimization process: each evaluation of ***G****_S_* requires *n* additional EM simulations of the antenna, *n* being the number of the antenna geometry variables. A comment should be made about the search region which is defined here as an interval −***d***^(*i*)^ ≤ ***x*** – ***x***^(*i*)^ ≤ ***d***^(*i*)^ (the inequalities are understood component-wise). The trust region size vector ***d***^(*i*)^ is adjusted using the standard rules based on the gain ratio [39]. This definition eliminates the need for variable scaling as the initial size vector ***d***^(0)^ is made proportional to the antenna parameter ranges. The gain ratio is defined as ρ = [*U(S*_11_(***x***^(*i*+1)^)) – *U(S*_11_(***x***^(*i*)^))]/[*U_S_(S_L_*(***x***^(*i*+1)^)) – *U_S_(S_L_*(***x***^(*i*)^))], and quantifies the actual versus (linear-model-) predicted objective function improvement. The iteration is considered successful, if ρ is positive, i.e., the improvement of the objective function was obtained, and the candidate design attained by solving (4) is accepted. The gain ratio value influences the trust region size ***d***^(*i*+1)^ in the next (***i*** + 1)th iteration in the following manner: ***d***^(*i*+1)^ is increased, if ρ > 0.75, and it is reduced, if ρ < 0.25.

### 2.3. Variable-Fidelity Simulation Models

**Figure 1:**
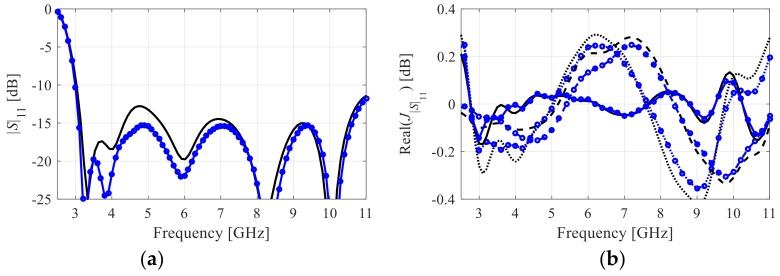
Exemplary reflection responses and reflection sensitivities of Antenna I of Section 3: (**a**) high-fidelity electromagnetic (EM) model reflection response (-o-o) and low-fidelity EM model response (—), (**b**) sensitivity with respect to selected antenna geometry parameters: high-fidelity EM model (⋅⋅o⋅⋅o⋅⋅, - o - o, - o - o)) and low-fidelity model (⋅⋅⋅⋅⋅, - - -, —).

In this work, following some of the surrogate-based optimization frameworks (e.g., [23]), a low-fidelity EM antenna model denoted as *S*_11.***c***_(***x***,*f*) is used to reduce the cost of sensitivity estimation. The low-fidelity model is normally obtained by manipulating the discretization density of the structure at hand, which results in faster simulation but also a certain loss of accuracy. Practical realization of this concept depends on the simulation environment utilized for implementing the computational model. For the examples considered in this work, CST Microwave Studio (Dassault Systemes, Vélizy, France) is used, where the major factor controlling the discretization density is LPW (lines per wavelength). By changing the value of this parameter, the overall number of mesh cells can be adjusted. The high-fidelity model is obtained upon performing grid convergence study, i.e., setting up LPW at the value that ensures stable simulation results (independent of small LPW alterations). The low-fidelity model is then obtained by selecting the lowest LPW that still ensures visual similarity of the antenna responses (at both low- and high-fidelity simulation levels). In particular, both models should properly account for the major features of the response, such as resonances [23]. An example of a well-chosen low-fidelity model can be found in Figure 1a. The accuracy loss is a reason that the low-fidelity model cannot be directly used as a replacement of the original model (unless appropriate corrections are made [23]), but correlations between the models can still be explored towards yielding computational advantages. Here, the low-fidelity model is used for the purpose of gradient estimation. Figure 1 shows low- and high-fidelity model responses for one of the antenna structures considered in Section 3 as well as the corresponding gradients (as functions of frequency). It can be observed that despite certain discrepancies between the responses, the gradients are quite well aligned, which indicates that the sensitivity estimated at the low-fidelity model level can be reliably utilized as a replacement of the high-fidelity ones within the gradient-based search procedures.

### 2.4. Proposed Optimization Framework

The core of the optimization framework proposed in this work is the trust-region gradient search algorithm of Section 2.2. Reduction of the computational cost of the optimization process is achieved by monitoring the changes of the antenna response gradients between iterations, using an appropriate metric. Small changes are an indication to omit the update of particular gradient components through finite differentiation. The second mechanism is to use the low-fidelity antenna model, instead of the high-fidelity one, for the purpose of sensitivity estimation.

As mentioned in Section 2.1, the problem of antenna matching improvement is considered here. The figure of interest is antenna reflection coefficient *S*_11_(***x***,*f*), which is a complex-valued function of antenna geometry parameter vector ***x*** and the frequency *f*. The gradient ***G****_S_* is a row vector, 1 × *n*. We denote by *G_k_* the *k*-th element of ***G****_S_*, *k* = 1, …, *n*. The gradient components are compared between subsequent iterations using the following metric

(6) $$d_{k}^{\left(i+1\right)}=\underset{f\in F}{mean}\left(2\cdot \frac{\left|G_{k}^{\left(i\right)}(f)\right|-\left|G_{k}^{\left(i-1\right)}(f)\right|}{\left|G_{k}^{\left(i\right)}(f)\right|+\left|G_{k}^{\left(i-1\right)}(f)\right|}\right)$$

where *G_k_*^(*i*)^(*f*) and *G_k_*^(*i*–1)^(*f*) refer to the *k*-th component of ***G****_S_* in the *i*th and (*i*–1)th iteration, respectively. Dependence of the gradient on the frequency *f* is shown explicitly. In (5), the averaging is performed over the frequency range of interest *f*.

The following notation is introduced for the purpose of subsequent considerations:

- ***d***^(*i*)^ = [*d*_1_^(*i*)^ … *d_n_*^(*i*)^]*^T^*—a vector of gradient difference factors (cf. (5)) used in the *i*-th iteration,
- *d*_min_^(*i*)^ = min{*k* = 1,…,*n*: *d_k_*^(*i*)^}, *d*_max_^(*i*)^ = max{*k* = 1,…,*n*: *d_k_*^(*i*)^}.

Furthermore, in the *i*-th iteration, a vector ***N***^(*i*)^ = [*N*_1_^(*i*)^ … *N_n_*^(*i*)^]*^T^* of the numbers of future iterations without FD is introduced. Its entries *N_k_*^(*i*)^ are determined by the affine conversion function

(7) $$N_{k}^{\left(i\right)}=〚N_{\max}+a^{\left(i\right)}(d_{k}^{\left(i\right)}-d_{\min}^{\left(i\right)})〛$$

where *a*^(*i*)^ = (*N*_max_ – *N*_min_)/(*d*_min_^(*i*)^ – *d*_max_^(*i*)^) and [[.]] denotes the nearest integer function. Where *N*_max_ and *N*_min_ denote the algorithm control parameters: the maximum and the minimum number of omitted FD calculations. For the *k*-th parameter, the function (6) describes the relation between the number of iterations without FD *N_k_*^(*i*)^ and the gradient difference coefficients *d_k_*^(*i*)^, which is based *N*_min_ and *N*_max_, respectively.

The vector ***N***^(*i*+1)^ is established as follows: if, for a given variable, FD was performed, the respective component *N_k_*^(*i*+1)^ is determined using (6), otherwise the previous number of iterations (from the *i*-th iteration) is decremented, i.e., *N_k_*^(*i*+1)^ = *N_k_*^(*i*)^ – 1. Clearly, for the variables characterized by the smallest gradient variation *N_k_*^(*i*)^ = *N*_max_ is assigned, and in consequence, the sensitivity update through FD is omitted for no more than *N*_max_ iterations. As for the difference factors *d_k_*^(*i*)^, their values are retained through all the iterations without FD. Thus, they are utilized to determine *d*_min_^(*i*)^ and *d*_max_^(*i*)^, and they are also involved in assessing the values of *N_k_*^(*i*)^ for other parameters.

**Figure 2:**
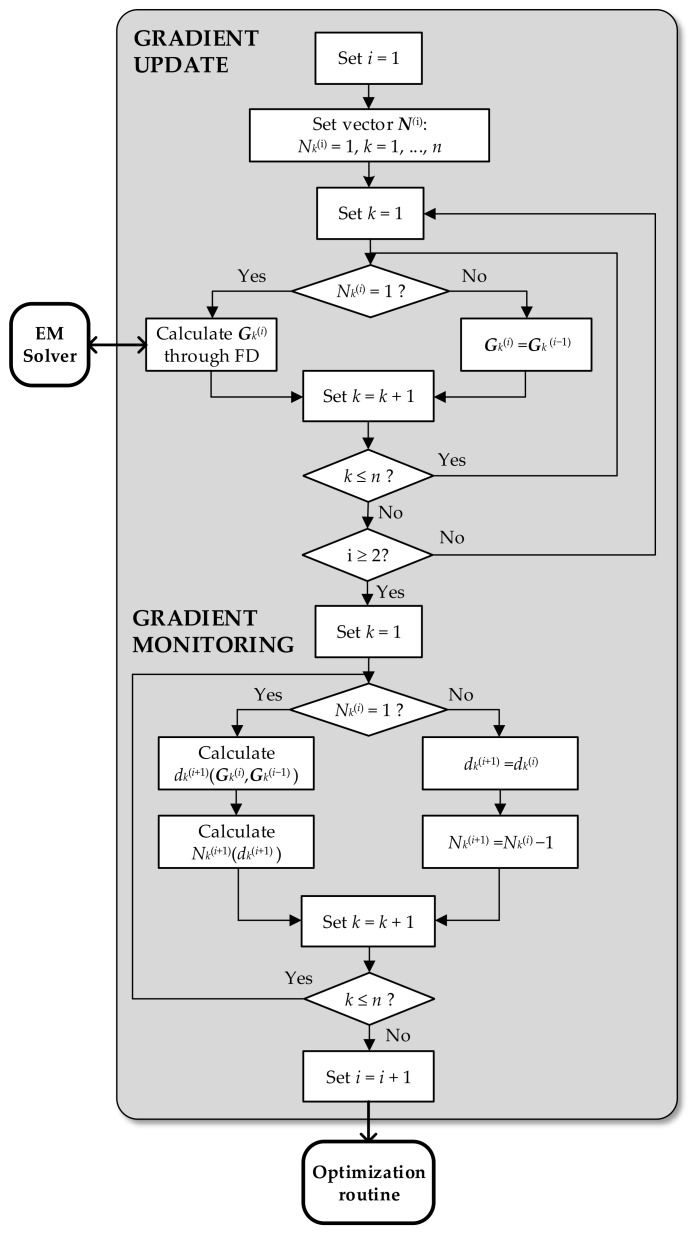
Flow diagram of the proposed sensitivity update routine with gradient change monitoring.

In the course of the reference algorithm of Section 2.2, the entire gradient is estimated using FD in each iteration. Whereas in the proposed optimization procedure, gradient ***G****_S_* is estimated solely through FD only in the first two iterations. Moreover, the gradient is exclusively computed using the low-fidelity EM model. In the succeeding iterations, the *k*th component *G_k_* of the gradient vector is established according to ***N***^(*i*+1)^: if *N_k_*^(*i*)^ = 1, FD is performed, otherwise the most recent value estimated with FD is kept. The above-described mechanisms allow for a significant reduction of the computational cost, expressed as the overall number of EM simulations, as it is verified by the results from the next section. This is achieved with an insignificant degradation of the design quality. Figure 2 shows the flow diagram of the algorithm. Table 1 highlights the main features of algorithms compared in the paper: the conventional trust region algorithm (single- and variable-fidelity setup), the pattern search algorithm (single-variability) and the proposed procedure (single- and variable-fidelity setup).

## 3. Verification Case Studies and Benchmarking

This section provides a comprehensive numerical validation of the proposed optimization framework. Three compact wideband antennas were utilized as test cases. Our algorithm is also benchmarked against the conventional trust-region algorithm of Section 2.2 as well as the accelerated version working entirely with the high-fidelity EM simulations. The contribution of both acceleration mechanisms into computational savings is discussed in detail.

### 3.1. Case Studies

**Figure 3:**
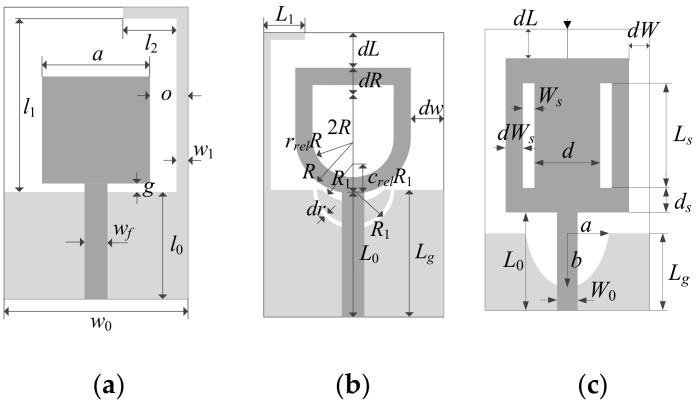
Benchmark antennas for verification of the proposed optimization algorithm: (**a**) Antenna I [40], (**b**) Antenna II [41], (**c**) Antenna III [42]. Ground plane marked using light gray shade.

The algorithm of Section 2.4 has been verified using three wideband antennas shown in Figure 3. The first structure, Antenna I, [40] was implemented on Taconic RF-35 substrate (*ε_r_* = 3.5, *h* = 0.762 mm). It is described by parameters *x* = [*l*_0_ *g a l*_1_ *l*_2_ *w*_1_ *o*]*^T^*, *w*_0_ = 2*o* + *a*, and *w_f_* = 1.7 mm. Antenna II [41] was also implemented on RF-35, and its independent geometry parameters were *x* = [*L*_0_ *dR R r_rel_ dL dw L_g_ L*_1_ *R*_1_ *dr c_rel_*]*^T^*. Antenna III [42] was implemented on FR4 substrate (*ε_r_* = 4.3, *h* = 1.55 mm). The design parameters were *x* = [*L_g_ L*_0_ *L_S_ W_S_ d dL d_S_ dW_S_ dW a b*]*^T^*. 

All three antennas were supposed to operate within the UWB frequency range of 3.1 GHz to 10.6 GHz. The computational models were implemented in CST Microwave Studio and evaluated using its time domain solver. The model setups were the following:

1. Antenna I: high-fidelity model (~800,000 mesh cells, simulation time 3.8 min = 230 s), low-fidelity model (~180,000 mesh cells, simulation time 77 s).
2. Antenna II: high-fidelity model (~830,000 mesh cells, simulation time 3.5 min = 210 s), low-fidelity model (~280,000 mesh cells, simulation time 88 s).
3. Antenna III: high-fidelity model (~520,000 mesh cells, simulation time 2.9 min = 176 s), low-fidelity model (~170,000 mesh cells, simulation time 79 s).

The computational models incorporated SMA connectors.

### 3.2. Experimental Setup

**Figure 4:**
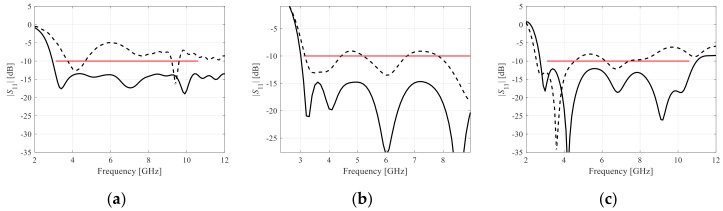
Initial (- - -) and optimized (—) responses of the antennas found using the proposed variable-fidelity algorithm, shown for the representative runs of the procedure: (**a**) Antenna I, (**b**) Antenna II, (**c**) Antenna III. Horizontal lines mark the design specifications.

The antennas of Figure 3 were optimized for best matching (cf. Section 2.1) within the UWB frequency range, using the proposed algorithm. For the sake of benchmarking, four other algorithms were compared: (1) the reference trust-region algorithm of Section 2.2 working with a high-fidelity model, (2) the pattern search algorithm [37], (3) the reference algorithm working with variable-fidelity models (low-fidelity model used for sensitivity estimation), and (4) the algorithm of Section 2.4 working with the high-fidelity model only. For each algorithm, ten runs were executed using random initial designs. The statistics of the results were used to determine the optimization process reliability as explained in Section 3.3. The algorithm of Section 2.4 was executed with the following values of its control parameters: *N*_min_ = 1, and *N*_max_ = 5. Table 1 gathers the relevant numerical data for the algorithm (1) through (4), as well as for the proposed algorithm (5). For illustration purposes, Figure 4 shows the initial and optimized antenna responses at the selected designs. The optimal geometry parameter values for the designs shown in Figure 4 are presented in Table 2.

### 3.3. Results and Benchmarking

The results obtained for the four considered algorithms are presented in Table 3: the reference algorithm with a high-fidelity model and the reference algorithm working with variable-fidelity models (Algorithm 1, Algorithm 3, respectively), as well as the algorithm of Section 2.4 with a high-fidelity model and its variable-fidelity modification (the Algorithms 4 and 5, respectively). The simulation time of the coarse model of Antenna I is three times shorter as compared to its high-fidelity model, whereas for Antennas II and III, it is 2.4 and 2.2 times shorter, respectively. During the optimization process, for the algorithms with variable-fidelity models (Algorithms 3 and 5), the expensive high-fidelity model is simulated only around 13 times for all the antennas. The results confirm that adopting variable-fidelity approach allows for achieving good design quality and significant cost savings for all antennas. In the case of the reference algorithm, the savings come from the use of the coarse model for estimating the gradient of the model response. This allows for reducing the overall optimization time for all antennas by a factor of around two. For Algorithm 5, the savings are also due to omitting the update of some part of the response gradients through finite differentiation. In that case, the reduction of the overall optimization time is as high as four times for the Antenna II (it is decreased from around 390 min for the reference algorithm with a high-fidelity model to around 96 min for the proposed algorithm in the variable-fidelity framework, i.e., Algorithm 5). It should also be mentioned that the pattern search algorithm is the most expensive one in the entire benchmark set and the quality of obtained designs is worse compared to both the reference algorithm and the accelerated versions.

## 4. Discussion

The results gathered in Table 3 indicate that the introduction of the variable-fidelity framework (Algorithm 3) allows for achieving approximately the same level of savings as the introduction of the accelerated algorithm of Section 2.4 (Algorithm 4). For these algorithms, the overall optimization cost is around 50 percent of that of the reference algorithm working at the level of high-fidelity EM model (Antennas I and II). In addition, the design quality is almost the same for Algorithms 3 and 4 for all benchmark cases. Combining both mechanisms as implemented in Algorithm 5 yields further savings accompanied by a minor reduction of the design quality for Antenna I and II (0.8 dB and 0.7 dB, respectively, w.r.t. Algorithm 1). In the case of Antenna III, the design quality degradation is higher, and it equals 2 dB. The most notable time savings of 75 percent are obtained for Antenna II. It should be emphasized that such considerable reduction of the optimization cost was achieved despite the fact that the time evaluation ratio between the high- and low-fidelity models is only between two and three for the considered antenna structures. In many cases (e.g., [24]), that ratio can be made much higher, consequently implying even more significant cost savings.

A remark on the accuracy-speed trade-off should be made. As it follows from Table 3, the proposed variable-fidelity framework allows for obtaining an acceptable solution quality much faster than the conventional trust-region procedure (Algorithm 1). Monitoring the objective function value throughout the optimization run reveals that, given the same optimization time (required by Algorithm 5 for its full convergence), Algorithm 1 arrives at a considerably higher maximum *S*_11_ within UWB frequency range: –9.9 dB (Antenna I), –12.2 dB (Antenna II) and –10.5 dB (Antenna III). The corresponding values for Algorithm 5 equal to: –11.1 dB, –14.2 dB and –11.9 dB, respectively. The above results indicate that the proposed algorithm produces better results than the reference algorithm assuming the same computational budget. On the other hand, although the results of the reference algorithm are slightly better (upon full convergence) than for the proposed method, the differences are minor from the practical point of view. The designer may be willing to sacrifice the accuracy to a certain extent having in return a considerable reduction of the computational cost.

## 5. Conclusions

The paper introduced a novel algorithm for accelerated gradient-based optimization of antenna input characteristics. By utilizing the two major mechanisms, i.e., suppressing finite-differentiation-based gradient updates for variables that exhibit stable sensitivity patterns, as well as utilizing coarse-discretization EM simulations for gradient estimation, considerable computational savings have been demonstrated over the conventional trust-region gradient algorithm. Furthermore, the reliability of the approach has been verified through statistical analysis involving multiple runs with random initial designs. The proposed framework can be applied to efficient design optimization of compact antennas, including devices for IoT applications.

## Figures and Tables

**Table 1:** Main features of the optimization procedures utilized in this work (including benchmarking).

Algorithm		Models Utilized	Operating Principle	Initial Gradient Estimation	Sensitivity Updates
1	Reference (high-fidelity)	high-fidelity	gradient	finite differentiation	finite differentiation
2	Pattern search [37]	high-fidelity	derivative-free (stencil based)	N/A	N/A
3	Reference (variable-fidelity)	high-fidelity low-fidelity	gradient	finite differentiation	finite differentiation
4	Alg. of Section 2.4 (high-fidelity)	high-fidelity	gradient	finite differentiation	sparse
5	Alg. of Section 2.4 (variable-fidelity)	high-fidelity low-fidelity	gradient	finite differentiation	sparse

**Table 2:** Optimal geometry parameter vectors for the representative algorithm runs of Figure 4.

Antenna	Geometry Parameter Values [mm]										
**I**	***l*_0_**	***g***	***a***	***l*_1_**	***l*_2_**	***w*_1_**	***o***				
	25.76	19.92	11.00	7.38	6.55	2.92	2.84				
II	*L*_0_	*dR*	*R*	*r_rel_*	*dL*	*dw*	*L_g_*	*L*_1_	*R*_1_	*dr*	*c_rel_*
	10.08	0.08	5.42	0.52	2.05	5.84	10.09	5.05	2.28	0.58	0.44
III	*L_g_*	*L*_0_	*L_s_*	*W_s_*	*d*	*dL*	*d_s_*	*dW _s_*	*dW*	*a*	*b*
	8.54	12.41	8.74	0.57	4.13	10.95	1.54	1.42	2.39	0.33	0.55

**Table 3:** Performance statistics of the proposed algorithm in the variable-fidelity framework.

Algorithm			Antenna I			Antenna II			Antenna III		
			Cost ^a^	max |*S*_11_|^b^ [dB]	Opt. time^c^ [*min*]	Cost ^a^	max |*S*_11_|^b^ [dB]	Opt. time^c^ [*min*]	Cost ^a^	max |*S*_11_|^b^ [dB]	Opt. time^c^ [*min*]
1	Reference (high-fidelity)		97.6	−11.9 [−9.9] ^d^	277.5	111.2	−14.9 [−12.2] ^d^	389.2	111.0	−13.9 [−10.5] ^d^	455.1
2	Pattern search		380.1	−11.9	1080.7	815.5	−14.5	2854.3	725.3	−12.5	2973.7
3	Reference	coarse	60.0	−11.8	119.8	97.2	−14.8	199.5	109.2	−13.7	199.2
	(variable-fidelity)	fine	13.9			13.4			14.4		
4	Alg. of Section 2.4 (high-fidelity)		46.1	−11.4	135.2	58.6	−14.7	205.0	68.7	−13.5	281.7
5	Alg. of Section 2.4	coarse	36.4	−11.1	90.5	45.4	−14.2	96.4	58.6	−11.9	133.9
	(variable-fidelity)	fine	14.5			12.7			14.3		

^a^ Number of EM simulations averaged over 10 algorithm runs (random initial points). ^b^ Maximum *|S*_11_| within UWB frequency range (averaged over 10 algorithm runs). ^c^ Overall optimization time. ^d^ Maximum |*S*_11_| within UWB frequency range (averaged over 10 algorithm runs) obtained by the reference algorithm within the overall optimization time of Algorithm 5.

## References

[1] Caytan O., Lemey S., Agneessens S., Vande Ginste D., Demeester P., Loss C., Salvado R., Rogier H. (2016). Half-mode substrate-integrated-waveguide cavity backed slot antenna on cork substrate. Ant. Wirel. Propag. Lett..

[2] Bashir U., Jha K.R., Mishra G., Singh G., Sharma S.K. (2017). Octahedron-shaped linearly polarized antenna for multistandard services including RFID and IoT. Trans. Ant. Propag..

[3] Zhang J., Shen Z. (2017). Compact and high-gain UHF/UWB RFID reader antenna. Trans. Ant. Propag..

[4] MMao Y., Guo S., Chen M. (2018). Compact dual-band monopole antenna with defected ground plane for Internet of things. Iet. Microw. Ant Propag..

[5] SShafique K., Khawaja B.A., Daniyal Khurram M., Maaz Sibtain S., Siddiqui Y., Mustaqim M., Chattha H.T., Yang X. (2018). Energy harvesting using a low-cost rectenna for Internet of Things (IoT) applications. IEEE Access.

[6] Lemey S., Castel T., Van Torre P., Vervust T., Vanfleteren J., Demeester P., Vande Ginste D., Rogier H. (2016). Threefold rotationally symmetric SIW antenna array for ultra-short-range MIMO communication. IEEE Trans. Ant. Propag..

[7] Narbudowicz A., Ammann M.J. (2018). Low-cost multimode patch antenna for dual MIMO and enhanced localization use. IEEE Trans. Ant. Propag..

[8] Su Z., Klionovski K., Bilal R.M., Shamim A. (2018). A dual band additively manufactured 3-D antenna on package with near-isotropic radiation pattern. IEEE Trans. Ant. Propag..

[9] Liu H., Cheng Y., Yan M. (2016). Electrically small loop antenna standing on compact ground in wireless sensor package. IEEE Ant. Wirel. Propag. Lett..

[10] Lizzi L., Ferrero F. (2015). Use of ultra-narrow band miniature antennas for internet-of-things applications. Electr. Lett..

[11] Dong Y., Choi J., Itoh T. (2017). Folded strip/slot antenna with extended bandwidth for WLAN application. IEEE Ant. Wirel. Propag. Lett..

[12] Li G., Huang Y., Gao G., Wei X., Tian Z., Bian L. (2017). A handbag zipper antenna for the applications of body-centric wireless communications and Internet of Things. IEEE Trans. Ant. Propag..

[13] Jha K.R., Bukhari B., Singh C., Mishra G., Sharma S.K. (2018). Compact planar multistandard MIMO antenna for IoT applications. IEEE Trans. Ant. Propag..

[14] Fernandez Pantoja M., Rubio Bretones A., Gomez Martin R. (2018). Benchmark antenna problems for evolutionary optimization algorithms. IEEE Trans. Ant. Propag..

[15] Chamaani S., Abrishamian M.S., Mirtaheri S.A. (2010). Time-domain design of UWB Vivaldi antenna array using multiobjective particle swarm optimization. IEEE Ant. Wirel. Prop. Lett..

[16] Darvish A., Ebrahimzadeh A. (2018). Improved fruit-fly optimization algorithm and its applications in antenna array synthesis. IEEE Trans. Ant. Prop..

[17] Goudos S.K., Siakavara K., Samaras T., Vafiadis E.E., Sahalos J.N. (2011). Self-adaptive differential evolution applied to real-valued antenna and microwave design problems. IEEE Trans. Ant. Propag..

[18] El-Hana Bouchekara H.R., Orlandi A., Al-Qdah M., de Paulis F. (2018). Most valuable player algorithm for circular antenna arrays optimization to maximum sideloba levels reduction. IEEE Trans. Ant. Propag..

[19] El Sabbagh M.A., Bakr M.H., Bandler J.W. (2006). Adjoint higher order sensitivities for fast full-wave optimization of microwave filters. IEEE Trans. Microw. Theory Tech..

[20] Ghassemi M., Bakr M., Sangary N. (2013). Antenna design exploiting adjoint sensitivity-based geometry evolution. Iet Microw. Ant. Prop..

[21] Toivanen J.I., Rahola J., Makinen R.A.E., Jarvenpaa S., Yla-Oijala P. (2010). Gradient-based antenna shape optimization using spline curves. Ann. Rev.Prog. Appl. Comp. Electromagnet..

[22] (2018). CST Microwave Studio, Ver. 2018.

[23] Koziel S., Ogurtsov S. (2014). Antenna Design by Simulation-Driven Optimization. Surrogate-Based Approach.

[24] Koziel S., Bekasiewicz A. (2016). Multi-Objective Design of Antennas Using Surrogate Models.

[25] Simpson T.W., Pelplinski J.D., Koch P.N., Allen J.K. (2001). Metamodels for computer-based engineering design: Survey and recommendations. Eng. Comput..

[26] Koziel S., Leifsson L. (2016). Simulation-Driven Design by Knowledge-Based Response Correction Techniques.

[27] Queipo N.V., Haftka R.T., Shyy W., Goel T., Vaidynathan R., Tucker P.K. (2005). Surrogate-based analysis and optimization. Prog. Aerosp. Sci..

[28] Chavez-Hurtado J.L., Rayas-Sanchez J.E. (2016). Polynomial-based surrogate modeling of RF and microwave circuits in frequency domain exploiting the multinomial theorem. IEEE Trans. Microwave Theory Tech..

[29] Angiulli G., Cacciola M., Versaci M. (2007). Microwave devices and antennas modelling by support vector regression machines. IEEE Trans. Magn..

[30] Kabir H., Wang Y., Yu M., Zhang Q.J. (2008). Neural network inverse modeling and applications to microwave filter design. IEEE Trans. Microwave Theory Tech..

[31] De Villiers D.I.L., Couckuyt I., Dhaene T. Multi-objective optimization of reflector antennas using kriging and probability of improvement. Proceedings of the 2017 IEEE International Symposium on Antennas and Propagation & USNC/URSI National Radio Science Meeting.

[32] Easum J.A., Nagar J., Werner D.H. Multi-objective surrogate-assisted optimization applied to patch antenna design. Proceedings of the 2017 IEEE International Symposium on Antennas and Propagation & USNC/URSI National Radio Science Meeting.

[33] Cheng Q.S., Koziel S., Bandler J.W. (2006). Simplified space mapping approach to enhancement of microwave device models. Int. J. RF Microwave Comput.-Aided Eng..

[34] Tu S., Cheng Q.S., Zhang Y., Bandler J.W., Nikolova N.K. (2013). Space mapping optimization of handset antennas exploiting thin-wire models. IEEE Trans. Ant. Propag..

[35] Su Y., Li J., Fan Z., Chen R. Shaping optimization of double reflector antenna based on manifold mapping. Proceedings of the 2017 International Applied Computational Electromagnetics Society Symposium (ACES).

[36] Koziel S., Unnsteinsson S.D. (2018). Expedited design closure of antennas by means of trust-region-based adaptive response scaling. IEEE Antennas Wirel. Propag. Lett..

[37] Koziel S. (2010). Computationally efficient multi-fidelity multi-grid design optimization of microwave structures. App. Comput. Electromagnet. Soc. J..

[38] Koziel S., Bandler J.W., Cheng Q.S. (2010). Robust trust-region space-mapping algorithms for microwave design optimization. IEEE Trans. Microwave Theory Tech..

[39] Conn A.R., Gould N.I.M., Toint P.L. (2000). Trust Region Methods.

[40] Koziel S., Bekasiewicz A. Low-cost multi-objective optimization of antennas using Pareto front exploration and response features. Proceedings of the IEEE International Symposium on Antennas and Propagation (APS-URSI).

[41] Alsath M.G.N., Kanagasabai M. (2015). Compact UWB monopole antenna for automotive communications. IEEE Trans. Ant. Prop..

[42] Haq M.A., Koziel S., Cheng Q.S. EM-driven size reduction of UWB antennas with ground plane modifications. Proceedings of the International Applied Computational Electromagnetics Society Symposium (ACES).

